# Wear Resistance, Color Stability and Displacement Resistance of Milled PEEK Crowns Compared to Zirconia Crowns under Stimulated Chewing and High-Performance Aging

**DOI:** 10.3390/polym13213761

**Published:** 2021-10-30

**Authors:** Simone Shah Abhay, Dhanraj Ganapathy, Deepak Nallaswamy Veeraiyan, Padma Ariga, Artak Heboyan, Pokpong Amornvit, Dinesh Rokaya, Viritpon Srimaneepong

**Affiliations:** 1 Department of Prosthodontics, Saveetha Dental College and Hospital, Saveetha Institute of Medical and Technical Sciences, Chennai 600077, India; simoneshah2293@gmail.com (S.S.A.); dhanaraj@saveetha.com (D.G.); drdeepaknallu@gmail.com (D.N.V.); padma@saveetha.com (P.A.); heboyan.artak@gmail.com (A.H.); 2 Department of Prosthodontics, Faculty of Stomatology, Yerevan State Medical University after Mkhitar Heratsi, Str. Koryun 2, Yerevan 0025, Armenia; 3 Golden Jubilee Medical Centre, Mahidol University, Nakon Pathom, Salaya 73170, Thailand; pokpong.amornvit@gmail.com; 4 Department of Clinical Dentistry, International College of Dentistry, Walailak University, Bangkok 10400, Thailand; 5 Department of Prosthodontics, Faculty of Dentistry, Chulalongkorn University, Bangkok 10330, Thailand

**Keywords:** PEEK, zirconia, crowns, biomaterials, biodegradation, CAD/CAM, chewing simulation, wear resistance, color stability, aging process, displacement resistance, dentistry, dental materials

## Abstract

Recently, polyetheretherketone (PEEK) has been introduced to the dental market as a high-performance and chemically inert biomaterial. This study aimed to compare the wear resistance, abrasiveness, color stability, and displacement resistance of zirconia and PEEK milled crowns. An ideal tooth preparation of a first maxillary molar was done and scanned by an intraoral scanner to make a digital model. Then, the prosthetic crown was digitally designed on the CAD software, and the STL file was milled in zirconia (CaroZiir S, Carol Zircolite Pvt. Ltd., Gujarat, India) and PEEK (BioHpp, Bredent GmbH, Senden, Germany) crowns using five-axis CNC milling machines. The wear resistance, color stability, and displacement resistance of the milled monolithic zirconia with unfilled PEEK crowns using a chewing simulator with thermocyclic aging (120,000 cycles) were compared. The antagonist wear, material wear, color stability, and displacement were evaluated and compared among the groups using the Wilcoxon–Mann–Whitney U-test. Zirconia was shown to be three times more abrasive than PEEK (*p* value < 0.05). Zirconia had twice the wear resistance of PEEK (*p* value < 0.05). Zirconia was more color stable than PEEK (*p* value < 0.05). PEEK had more displacement resistance than zirconia (*p* value < 0.05). PEEK offers minimal abrasion, better stress modulation through plastic deformation, and good color stability, which make it a promising alternative to zirconia crown.

## 1. Introduction

Wear and tribological properties are important in dentistry [1,2]. Progressive wear of the dental materials from the tooth and restorations occurs due to excessive mechanical and chemical processes such as mastication, erosion, and abrasion [3,4]. Hence, abrasion resistance, as well as abrasiveness, are important properties of restorative materials [5]. Recently, restorative materials with high wear resistance are being developed in the clinical scenario [6]. Although there are various in vitro studies on the wear and abrasiveness of the restorative materials, there are fewer studies representing the simulated oral conditions. Furthermore, the compressive strength and displacement resistance play an important role in the selection of restorative material, which can be studied from a universal testing machine [7].

The discoloration is another significant parameter that needs to be studied extensively. This value of the color stability of the restorative material is evaluated over a prolonged period using a spectrophotometer [8]. The surface roughness and surface free energy significantly influence the color stability of the material. The color stability is also affected by the thermocyclic aging of the restorative material. This leads to a color change from chroma change and hue difference [9].

**Figure 1:**
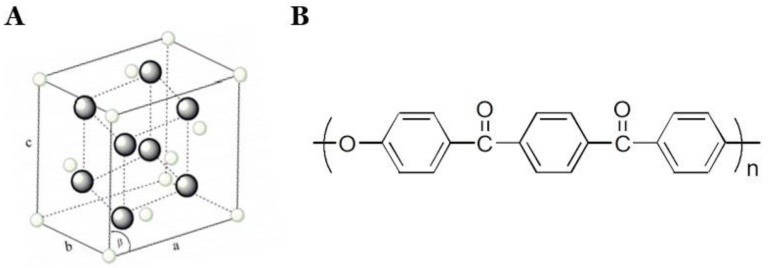
Structure of zirconia (**A**) and polyetheretherketone (PEEK) (**B**). Modified from [13].

Zirconia (Zirconium oxide, ZrO_2_) is currently the most used tooth-colored restorative material in dentistry (Figure 1A). It has excellent esthetics, good biocompatibility, and superior mechanical properties to metals. The wear resistance of zirconia is higher than metal, and thus, it is even being used to fabricate implants and implant abutments due to its high strength [10]. Fully contoured monolithic zirconia is made through CAD/CAM technology completely. This eliminates the dental laboratory work in the manual fabrication of the crowns, thus increasing the precision [11,12].

Polyaryletherketone (PAEK) consists of polyetheretherketone (PEEK) and polyetherketoneketone (PEKK), and it was recently introduced in dentistry [14]. Recently, PEEK and PEKK were introduced to the dental market as high-performance and chemically inert biomaterials [14,15]. With its extremely low potential to trigger an allergy, PEEK has very few reported systemic immune responses after intraoral insertion [16]. PEEK can become an alternative to conventional and well-investigated veneering ceramics and denture base resin materials with low discoloration and improved mechanical properties (Figure 1B) [17]. PEEK-based materials are being used along with or instead of polymethyl methacrylate (PMMA) and resin composite materials in both removable and fixed dental prostheses. PEEK also has a low modulus of elasticity compared to the bone, which allows for a better absorption of functional stress by deformation. This acts as a great advantage over the ceramic materials used. PEEK also shows low abrasive wear of the enamel in comparison to ceramic materials. Carbon and glass fiber variants of PEEK have higher flexural strength and color stability. PEEK is highly color stable in comparison to PMMA and composite resin materials. To improve its esthetic properties, various manufacturers have added composite resins and titanium dioxide into the PEEK material. PEEK exhibits excellent chemical resistance due to its chemical structure; thus, it can withstand high temperatures without significant degradation [18,19].

There has been a vast development in digital technologies in dentistry including 3D scanning, designing, and 3D/4D/5D printing [20,21,22,23,24]. The zirconia, metal, PEEK, and PMMA prosthetic crowns can be digitally designed and milled using milling machines. Milling machines are available as three-axis, four-axis, and five-axis milling. The accuracy of the milling machine determines the final milled output. IMES icore 350i is a five-axis milling machine that is one of the standard machines for milling various prosthetic materials [25,26]. High-quality milling machines with standardized protocols that are used globally produced highly accurate crowns.

PEEK can be a promising alternative to titanium and zirconium to use in various clinical situations in dental practice due to its high-quality mechanical properties such as favorable elastic modulus, strength, rigidity, and light weight [15,27,28,29]. The PEEK prostheses have shown better properties than the unmodified form of PEEK. It can be modified with various biomaterials to produce a composite with improved properties for various biomedical applications [27,30,31,32,33,34,35,36,37], such as carbon fiber-reinforced PEEK (CFR-PEEK) [32,34,35], resin-PEEK [36], nilinated-poly (ether ether ketone) (AN-PEEK), and AN-PEEK/f-CNOs composite thin films [30].

Although PEEK is widely used at present, there are very limited studies that compared the mechanical properties of PEEK as fixed prostheses with other materials such as zirconia. In addition, no study compared the mechanical properties of zirconia and PEEK using five-axis milling. Thus, this study aimed to compare the wear resistance, abrasiveness, color stability, and displacement resistance of PEEK and zirconia milled crowns using a chewing simulator with thermocyclic aging.

## 2. Materials and Methods

The study was approved by the Scientific Review Board of Saveetha University (SRB/SDMDS09/18/PROSTHO/001). A total of 24 crowns were made, which were divided into two different groups based on two different materials taking reference to sample size from previous studies [38,39]. Group 1 (*n* = 12) consisted of milled zirconia crowns (CaroZiir S, Carol Zircolite Pvt. Ltd., Gujarat, India) and Group 2 (*n* = 12) consisted of milled PEEK crowns (BioHpp, Bredent GmbH, Senden, Germany). Each group was divided into 3 subgroups, as shown in Table 1. Group 1 consists of (1a) zirconia control, (1b) zirconia thermocycled, and (1c) zirconia worn and thermocycled, and Group 2 consists of (2a) PEEK control, (2b) PEEK thermocycled, and (2c) PEEK worn and thermocycled.

### 2.1. Die Preparation

Tooth preparations were done on a first maxillary molars of the Typodont model (PER5001-UL-SCP-AK-28, Nissin Dental, Kyoto, Japan) using a taper flat end bur for the buccal and palatal surfaces and were replicated into multiple epoxy resin dies (Acculite Die Epoxy 8000 Fast, Henry Schein Inc., New York, NY, USA). The tooth preparations had 1.5 mm shoulder margins ideal for an all-ceramic restoration with a clearance of 2 mm occlusally according to the previous study [40].

### 2.2. Designing and Milling of Zirconia and PEEK Crowns

The teeth were scanned in with an intraoral scanner (Trios 3, 3Shape, Copenhagen, Denmark), and the first maxillary molar crown was digitally designed on the CAD software. From STL files, zirconia and PEEK crowns were created and milled using a 5-axis computerized numeric control (CNC) milling machine (IMES iCore 350i, imes-icore GmbH, Eiterfeld, Germany) [41,42]. The zirconia crowns were sintered at 1450 °C for 60 min [43]. 

Then, the digitally fabricated zirconia and PEEK crowns were polished with polishing paste and a rubber wheel according to the manufacturer’s protocol [44,45]. The crown sizes were 11.8 mm in length, 7 mm in width, 2 mm in thickness occlusal, and 1.5 mm thickness at the margin, the middle, and cervical portion of the crown [40]. These precise measurements were made while designing the crowns in the CAD software (3 Shape, Düsseldorf, Germany).

### 2.3. Cementation of the Crowns onto the Die

After the fabrication, the crowns were stored at room temperature (25 °C) for at least 1 day, and any possible crack formations were examined. Then, the crowns were sandblasted with 100 µm at 1 bar pressure for 10 s. They were conditioned with 2 layers of bonding agents (ScotchBond Universal, 3M ESPE, Seefeld, Germany) and then cured for 20 s using curing light (Elipar Deepcure, 3M ESPE, Seefeld, Germany) as recommended by the manufacturer [46,47]. Then, the crowns were permanently cemented onto the epoxy resin dies using luting resin cement (RelyX U200, 3M ESPE, Seefeld, Germany) in the isolated field.

### 2.4. Chewing Simulation

The crown samples were prepared in the resin die and mounted with fast-setting high-strength low-expansion plaster (WhipMix Mounting Plaster, Ivoclar Vivadent AG, Schaan Fürstentum, Liechtenstein). The steatite antagonists (Steatite, SD Mechatronik, Munich, Germany) were used to mimic enamel and mounted using acrylic resin onto the aluminum mounts. The steatite balls were of 4 mm width and length. They were kept in cusp to fossa occlusion with the zirconia and PEEK crowns.

The chewing simulator (CS-4, SD Mechatronik, Munich, Germany) had 4 testing chambers within a thermocycling chamber. They have 2 moving parts: the vertical bar (Z-axis) and the horizontal table (X-axis). The samples were mounted onto the table, which could move back and forth. The antagonists were connected to the vertical bar and moved vertically. The load of 5 kg weights was created and applied to the samples by the antagonists. The antagonist samples of 4 mm diameter (Steatite, SD Mechatronik, Munich, Germany) were held by the vertical bar and the horizontal bar held the force sensors in order to evaluate the force applied at each cycle. The load was added to each rod to increase the masticatory load on each sample to simulate the oral environment. Thermocycling was done from 4 to 60 °C and each cycle consisted of one cold and one hot cycle, and the filling time of the chamber was kept for 12 s [48,49]. Chewing stimulation was done for 120,000 cycles [50,51].

### 2.5. Surface Wear of Samples Using a Laserscanner

**Figure 2:**
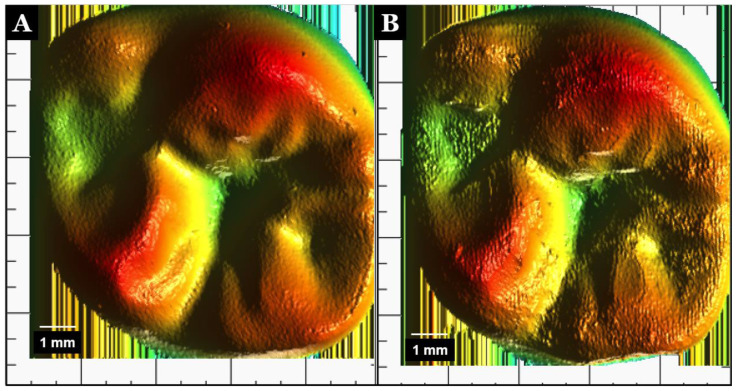
Color images of crowns after wear. Zirconia (**A**) and PEEK (**B**).

The zirconia and PEEK samples were pretested by a 3D laserscanner (LAS-20, SD Mechatronik, Munich, Germany), and the color graph was obtained. The surface wear of the samples was analyzed and recorded following the chewing simulator cycles with the thermocycling process. The vertical sensor resolution is 0.8 µm; thus, fine movements and cracks were detected. The samples were inserted onto the self-centering mount. The measurement fields were defined by a built-in camera, and the images were exported as jpeg files to be evaluated (Figure 2).

### 2.6. Color Stability Using a Digital Spectrophotometer 

A digital spectrophotometer (Vita Easyshade Compact, Vita Zahnfabrik, Bad Säckingen, Germany) has been used to determine the precise and reliable shade matching for natural teeth and ceramic restorations. It was also used to evaluate the color stability of the crowns before and after thermocycling and the chewing simulator. The digital spectrophotometer measured the wavelength range from 400 to 700 nm and used LED technology, which was unaffected by the environment.

### 2.7. Displacement of the Crown Using a Universal Testing Machine

**Figure 3:**
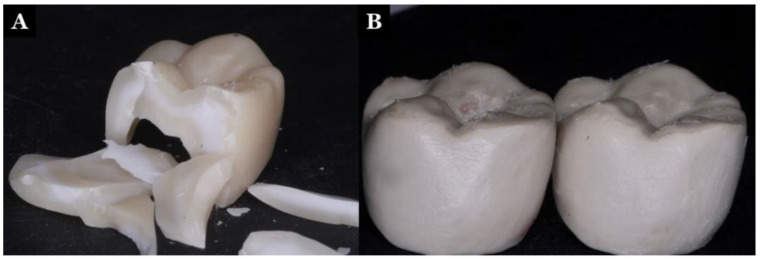
Displaced and fractured zirconia crown (**A**) and displaced and flattened cusp of the PEEK crown (**B**).

A universal testing machine (Instron 5566, *Instron* Ltd., Buckinghamshire, UK) capable of dynamic and static testing was used to evaluate the displacement of the crowns. The samples were mounted on the epoxy resin dies (Acculite Die Epoxy 8000 Fast, Henry Schein Inc., New York, NY, USA) and placed onto the platform of the universal testing machine. The zirconia and PEEK crowns were tested before and after the wear test and thermocycling process. They were subjected to a load of 2500 Ncm^−1^. The displacement of the material was observed in mm^2^ (Figure 3).

### 2.8. Statistics

All the data obtained were analyzed using the SPSS (SPSS 20, SPSS Inc., Chicago, IL, USA). Normality tests for the antagonist wear (mm^2^), material wear (mm^2^), color stability (ΔE), and displacement (mm^2^) were done using the Kolmogorov–Smirnov test, Levene test, and Shapiro–Wilk test, and the data were not normally distributed. As the variables were not normally distributed, non-parametric tests using Wilcoxon–Mann–Whitney *U*-tests were used to compare the values among the groups. The significant level was set at *p* value = 0.05.

## 3. Results

Wear of the steatite antagonist caused by zirconia compared to PEEK showed a significant difference (*p* < 0.001), as the zirconia caused three times more antagonist wear than PEEK, as shown in Table 2.

Similarly, the zirconia crowns were more wear-resistant than PEEK crowns over 120,000 cycles in the chewing simulator (*p* < 0.001), as shown in Table 3. PEEK crowns showed two times more wear than zirconia crowns (*p* < 0.001).

**Figure 4:**
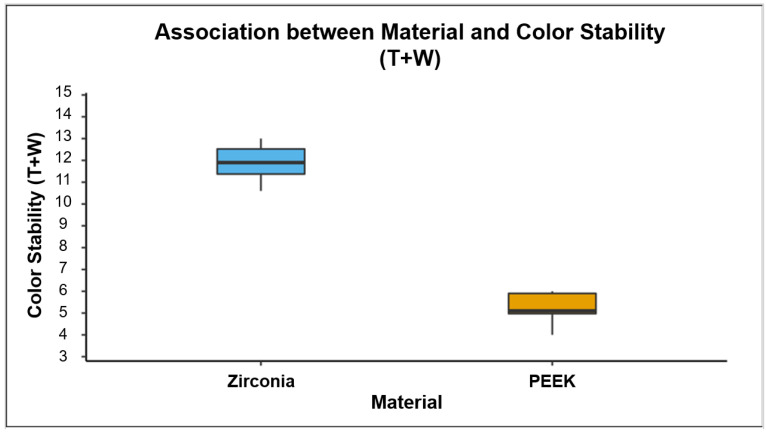
The Box-and-Whisker plot shows the distribution of color stability of thermocycled and worn zirconia and PEEK crowns. The color stability of thermocycled and worn zirconia was significantly better than that of the PEEK crowns (*p* < 0.001).

The ΔE values of color stability significantly decreased (*p* < 0.001) when the zirconia and PEEK crowns underwent wear and aging in the chewing simulator, as shown in Table 4. In addition, the ΔE value showed a significant difference among the control, thermocycled, and thermocycled and worn groups of both zirconia and PEEK crowns (*p* < 0.001). The color stability of thermocycled and worn zirconia was better than that of PEEK crowns (*p* < 0.001) (Figure 4).

**Figure 5:**
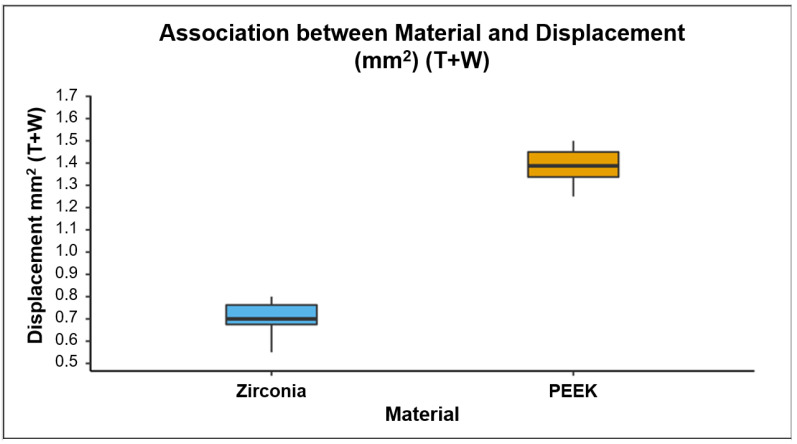
The Box-and-Whisker plot shows the distribution of displacement (mm^2^) of thermocycled and worn zirconia and PEEK crowns. The displacement resistance of thermocycled and worn PEEK was higher than that of the zirconia crowns (*p* < 0.001).

The resistance to displacement of the zirconia and PEEK materials decreased significantly (*p* < 0.001) following thermocycling and wear in the chewing simulator (Table 5). It also showed a significant difference between the control, thermocycled, and thermocycled and worn groups in both zirconia and PEEK crowns (*p* < 0.001). Furthermore, PEEK crowns showed significantly (*p* < 0.001) more resistance to displacement than zirconia crowns following thermocycling and wear in the chewing simulator (Figure 5).

## 4. Discussion

Although various new biomaterials are introduced for biomedical applications, PEEK has been widely used for dental applications [52,53,54,55,56,57]. The abrasion resistance, color stability, and displacement resistance of the restorative material are the important properties of restorative materials [5,6,7,9]. In this study, these properties are studied and compared between milled zirconia and PEEK crowns. It was found that the crowns made of zirconia produced three times more antagonist wear (*p* value < 0.05), maintained higher color stability (*p* value < 0.05), and offered the least displacement (*p* value < 0.05) compared with crowns made of PEEK. These findings are similar to the previous studies [58,59,60,61].

This study used standardized steatite balls, since it is a standard antagonist used for wear simulation studies [62]. The modulus of elasticity of this material is close to that of enamel. Steatite had good thermal conductivity and good thermal shock resistance; thus, it could withstand the constant force and thermocycling process in the chewing simulator. Thus, the steatite balls did not affect the results in this present study, as they had a modulus of elasticity close to the enamel and were used as antagonists in both study groups. In addition, zirconia and PEEK both showed satisfactory results [63,64,65]. Hence, it can be assumed that the properties of these two materials in this study are similar to the materials evaluated from the previous study by Daou [66].

In this study, the thermocycling temperature in the chewing simulator was simulated to match the oral environment. It was variable between −10 and 60 °C [40]. This was a standardized protocol used in the previously mentioned studies. Since this standardized protocol was used, it did not have confounding effects in the present study. A standardized axis origin was established for all the samples, and a 2 mm radius was allowed for movement in the x, y, and z-axis. Every sample went through the same amount of cycles (120,000 cycles) with a constant force of 200 Ncm^−1^ [67]. The movement of antagonists established was buccal to lingual with a thermal cyclic loading time of 12 s [40]. The occlusion was established as a cusp to the fossa in relation to the steatite balls occluding with the central fossa of the crowns, as mentioned in the previous studies [40,68]. The chewing simulator was standardized according to the manufacturer for the present study. Thus, it can be eliminated as one of the confounding factors.

The process of sintering was carried out at 1450 °C for 60 min, as recommended by the previous studies [69,70]. This was a standardized protocol followed for the sintering of zirconia [70]. However, this protocol is known to have a certain amount of shrinkage. This factor could affect the results of the study; however, the final measurements of the crowns were made, and this did not seem to affect the result of the study. 

Zirconia can be polished or glazed, and it has been shown that polishing produces minimal wear compared to glazed zirconia [44,71,72,73]. So, we did polishing for the zirconia crowns instead of glazing [74,75,76]. PEEK was polished using a rubber wheel and polishing paste as recommended in previous studies [33]. Even polishing was carried out for both the materials; thus, this factor could have a limited effect on the results. This study only evaluated the wear resistance and did not consider the hardness or scratch resistance. Hence, these factors may be confounding factors. 

In this study, strict environment and lighting protocol were maintained as reported in the previous study [71]. A calibrated Vita Easyshade guide digital spectrophotometer has been widely used for color assessment of various composite and porcelain materials [77,78]. The measurement made was independent of the surrounding light and heat present; thus, this parameter had limited or almost no effect on the final color stability of the zirconia and PEKK crowns.

The displacement in millimeters at a force of 2500 kN was evaluated for the PEEK and zirconia samples [79]. This was carried out using an Instron Electropuls E3000 all-electric dynamic testing machine. Since the machine was calibrated every time before testing, it could have little or no effect on the results of the present study.

The evaluation of the wear can be observed using various contact and non-contact profilometers such as True Definition (TD) from the 3M ESPE and 3D laserscanner (LAS-20) from SD MEchatronik [50]. Although many studies have mentioned that True Definition is more accurate than 3D laserscanner for the evaluation of wear, no statistical significance between both the machines was found [50,80], and the 3D laserscanner presents optimal accuracy [81]. In this present study, we used the 3D laserscanner to evaluate the wear pattern in two materials.

There are certain limitations to this study. Enamel was not used as an antagonist due to errors in standardization, the wear pattern was simulated only in the buccolingual direction, the circular movement of mastication was not replicated, and distilled water was used in the simulator instead of artificial saliva. Various confounding factors in this study can be the type of antagonist used, thermocycling process, characteristics of the chewing simulator, accuracy of the milling machine, and effect of the environment on the color stability. Although the surface wear can be studied from various techniques such as scanning electronic microscope (SEM) and energy-dispersive X-ray spectroscopy (EDS) [2], we used only the 3D laserscanner due to its availability and limited time.

The present report tested only wear, color stability, and displacement. It could be interesting in the future to test and compare PEEK materials also for flexural strength [82], synergic potential [83], and biocompatibility using cell culture [52,84]. In addition, the future scope of this research is a long-term split-mouth double-blinded randomized controlled clinical trial to evaluate the wear pattern and color stability of the zirconia and PEEK crowns.

## 5. Conclusions

Zirconia is currently the most used tooth-colored restorative material, and PEEK is used as an alternative to zirconium in dentistry. Within the limitation of this study, the crowns made of zirconia produced three times more antagonist wear, maintained higher color stability, and offered the least displacement compared with crowns made of PEEK. The PEEK crowns showed minimal abrasion, better stress modulation through plastic deformation, and good color stability, which makes it a promising alternative to zirconia for fabrication of the crown. The clinician can choose the material for the fabrication of dental prostheses according to the application considering its properties, advantages, and limitations.

## Figures and Tables

**Table 1:** Details of zirconia and PEEK restorations used in this study.

Groups	Prosthetic Crowns (*n* = 24)	
	Zirconia (*n* = 12)	PEEK (*n* = 12)
Control (C)	zirconia control (1a) (*n* = 4)	PEEK control (2a) (*n* = 4)
Thermocycled (T)	zirconia thermocycled (1b) (*n* = 4)	PEEK thermocycled = (2b) (*n* = 4)
Thermocycled + Worn (T + W)	zirconia worn and thermocycled (1c) (*n* = 4)	PEEK worn and thermocycled (2c) (*n* = 4)

**Table 2:** Results of antagonistic wear produced by zirconia and PEEK crowns.

Antagonist Wear (mm^2^) (Post-Wear)	Material		Wilcoxon–Mann–Whitney *U* Test	
	Zirconia	PEEK	W	*p* Value
Mean (SD)	6.17 (0.92)	2.50 (0.60)	144.00	<0.001 *
Median (IQR)	6.33 (0.94)	2.62 (1)		
Range	4.25–7.5	1.5–3.5		

SD = standard deviation, IQR = interquartile range. * Significant differences at *p* value < 0.05.

**Table 3:** Results of wear of the zirconia and PEEK crowns.

Material Wear (mm^2^) (Post-Wear)	Material		Wilcoxon–Mann–Whitney *U*-Test	
	Zirconia	PEEK	W	*p* Value
Mean (SD)	1.68 (0.49)	3.75 (0.89)	0.500	<0.001 *
Median (IQR)	1.68 (0.56)	3.5 (1.12)		
Range	1–2.5	2.5–5.5		

SD = standard deviation, IQR = interquartile range. * Significant differences at *p* value < 0.05.

**Table 4:** Results of the color stability of the zirconia and PEEK crowns.

Color Stability	Crown Materials				*p* Value
	Zirconia		PEEK		
	Mean (SD)	Median (IQR)	Mean (SD)	Median (IQR)	
Control (C)	17.71 (0.68)	17.85 (0.62)	9.58 (0.51)	9.50 (0.65)	<0.001 *
Thermocycled (T)	14.72 (0.85)	14.65 (1.12)	6.23 (0.40)	6.20 (0.58)	<0.001 *
Thermocycled + Worn (T + W)	11.88 (0.81)	11.90 (1.15)	5.28 (0.60)	5.10 (0.93)	<0.001 *
Friedman Test (*p* value)	<0.001 *		<0.001 *		

SD = standard deviation, IQR = interquartile range. * Significant differences at *p* value < 0.05.

**Table 5:** Results of the change in displacement of the zirconia and PEEK crowns.

Displacement (mm^2^)	Material				*p* Value
	Zirconia		PEEK		
	Mean (SD)	Median (IQR)	Mean (SD)	Median (IQR)	
Control	0.73 (0.05)	0.72 (0.06)	1.90 (0.18)	1.90 (0.20)	<0.001 *
Thermocycled (T)	0.45 (0.11)	0.44 (0.14)	1.49 (0.09)	1.48 (0.11)	<0.001 *
Thermocycled + Worn (T + W)	0.70 (0.09)	0.70 (0.09)	1.39 (0.08)	1.39 (0.11)	<0.001 *
Friedman Test (*p* value)	<0.001 *		<0.001 *		

SD = standard deviation, IQR = interquartile range. * Significant differences at *p* value < 0.05.

## Data Availability

The data presented in this study are available on request from the corresponding authors.
